# Differences in the Gene Expression Profiles of Slow- and Fast-Forming Preinduced Pluripotent Stem Cell Colonies

**DOI:** 10.1155/2015/195928

**Published:** 2015-04-07

**Authors:** Sujin Kwon, Jung Sun Park, Byungkuk Min, Yong-Kook Kang

**Affiliations:** ^1^Development and Differentiation Research Center, KRIBB, 111 Gwahak-ro, Yuseong-gu, Daejeon 305-806, Republic of Korea; ^2^Department of Functional Genomics, University of Science and Technology (UST), 113 Gwahang-ro, Yuseong-gu, Daejeon 305-333, Republic of Korea

## Abstract

Induced pluripotent stem cells (iPSCs) are generated through a gradual process in which somatic cells undergo a number of stochastic events. In this study, we examined whether two different doxycycline-inducible iPSCs, slow-forming 4F2A-iPSCs and fast-forming NGFP-iPSCs, have equivalent levels of pluripotency. Multiplex reverse-transcriptase PCR generated gene expression profiles (GEPs) of 13 pluripotency genes in single initially formed-iPSC (if-iPSC) colonies of NGFP and 4F2A group. Assessment of GEP difference using a weighted root mean square deviation (wRMSD) indicates that 4F2A if-iPSCs are more closely related to mESCs than NGFP if-iPSCs. Consistently, *Nanog* and *Sox2* genes were more frequently derepressed in 4F2A if-iPSC group. We further examined 20 genes that are implicated in reprogramming. They were, overall, more highly expressed in NGFP if-iPSCs, differing from the pluripotency genes being more expressed in 4F2A if-iPSCs. wRMSD analysis for these reprogramming-related genes confirmed that the 4F2A if-iPSC colonies were less deviated from mESCs than the NGFP if-iPSC colonies. Our findings suggest that more important in attaining a better reprogramming is the mode of action by the given reprogramming factors, rather than the total activity of them exerting to the cells, as the thin-but-long-lasting mode of action in 4F2A if-iPSCs is shown to be more effective than its full-but-short-lasting mode in NGFP if-iPSCs.

## 1. Introduction

Differentiated cells can be reprogrammed to pluripotent cells through viral introduction of the four transcription factors Oct4, Sox2, Klf4, and c-Myc [1–6]. However, this approach inevitably engenders random viral infection and random integrations with varying copy number into multiple loci of the genome of the induced pluripotent stem cell (iPSC). The resulting genetic heterogeneity complicates the analysis and interpretation of crucial molecular events governing somatic cell reprogramming. To circumvent this limitation, “secondary” reprogramming systems have been developed [7–11].

There are two different kinds of “secondary” systems reported in mice. One uses iPSCs that have been made with doxycycline- (Dox-) inducible lentiviral vectors expressing the four reprogramming factors. These clonal “primary” iPSCs are injected into blastocysts to produce chimeric fetuses from which genetically homogeneous, iPSC-derived mouse embryonic fibroblasts (mEFs) are obtained [10]. The other system relies on “reprogrammable” mice [12, 13]. These transgenic mice harbor a Dox-inducible, single polycistronic cassette encoding the four reprogramming factors [14] in the 3′-untranslated region of the collagen type I alpha 1 gene (*Col1a1*) [15]. Upon exposure to Dox, cells derived from these two systems are transformed into “secondary” iPSCs, without additional factor delivery.

The generation of iPSCs from somatic cells is a gradual process during which a number of stochastic epigenetic events take place as the cells are reprogrammed to intermediate iPSCs, or “pre-iPSCs” [16–20], and finally to fully reprogrammed, stabilized iPSCs [16, 21]. Intermediate iPSCs are cells that have acquired pluripotency to certain degree but nevertheless still retain an epigenetic memory of their cell type of origin. Those intermediate iPSCs of different origins and derivations can form colonies that are indistinguishable in morphology from stabilized iPSC colonies and mouse embryonic stem cell (mESC) colonies but it is unknown whether they have similar biochemical nature in them or contain variable degrees of pluripotency with different levels of epigenetic traces left on the genome. In this study, we examined whether the two different groups of early iPSCs obtained from different secondary systems have similar levels of pluripotency. For this, we assessed to what extent the two iPSC groups are different from each other in gene expression profiles (GEPs) of two different groups of genes that are implicated in pluripotency and reprogramming using the weighted root mean square deviation method (wRMSD) we recently developed [22].

## 2. Methods

### 2.1. Ethics Statement

This study was carried out in strict accordance with the recommendations in the Guide for the Care and Use of Laboratory Animals of the National Livestock Research Institute of Korea. The protocol was approved by the Committee on the Ethics of Animal Experiments of Korea Research Institute of Bioscience and Biotechnology. All the surgery was performed under sodium pentobarbital anesthesia, and all efforts were made to minimize suffering.

### 2.2. Chimera Formation and mEF Isolation

NGFP1 iPSC line was purchased from Stemgent (Cambridge, MA). NGFP1 iPSCs were injected into BDF1 (C57/B6 × DBA/1) hybrid blastocysts (94–98 h after hCG injection). A flat-tip microinjection pipette with an internal diameter of 12–15 mm was used for iPSC injection. About ten cells were placed into the blastocyst cavity and the injected blastocysts were immediately transferred to recipient females. Twelve to 15 blastocysts were transferred to the uterine horn of pseudopregnant BDF1 mouse at 2.5 days postcoitum.

NGFP-mEFs were isolated as described before [10]. Briefly, chimeric embryos were collected at E13.5, and the head and internal organs were removed. The remaining carcass was physically dissociated and incubated in trypsin at 37°C for 20 min. Dissociated cells were resuspended in MEF media containing puromycin (2 *μ*g/mL) and expanded for two passages before freezing or plating on Dox-containing medium for reprogramming experiments. For isolation of 4F2A-mEFs, 4F2A transgenic mouse (*Gt*(*ROSA*)26*Sor* ^*tm*1(*rtTA*^∗^*M*2)*Jae*^ *Col*1*a*1^*tm*3(*tetO*-*Pou*5*f*1,-*Sox*2,-*Klf*4,-*Myc*)*Jae*^/*J*) was purchased from Jackson Laboratory (stock number 011004). E13.5 fetuses were obtained from 4F2A homozygote intercross and 4F2A-mEFs were obtained from the carcass and expanded for two passages before freezing or plating on Dox-containing medium for reprogramming.

### 2.3. Reprogramming and Colony Picking

For reprogramming, NGFP- and 4F2A-mEFs (p3-4) were plated on Geltrex-coated dishes at 2 × 10^4^ cells/cm^2^ in MEF medium (DMEM supplemented with 10% FBS, 0.1 mM nonessential amino acids (NEAA), and 2 mM Glutamax). The following day, doxycycline (Sigma-Aldrich; 2 *μ*g/mL) treatment was initiated and cells were cultured in MEF medium for an additional day before culture in reprogramming initiation medium (RepM-Ini; knock-out DMEM supplemented with 10% knock-out serum replacer, 5% FBS, 0.1 mM NEAA, 2 mM Glutamax, and 0.055 mM *β*-mercaptoethanol) for indicated periods. From the induction culture where initial colonies were growing, single colonies reaching a certain size (2500–3000 cells per colony) were picked with a pipette tip and were separately transferred to PCR tube. The remaining culture after colony picking was discarded without passage and further maintenance. For counting the number of colony, NGFP and 4F2A if-iPSC colonies were first stained for alkaline phosphatase (AP) according to the manufacturer's manual.

### 2.4. RT-PCR and Multiplex PCR

Total RNAs were prepared from J1 mESCs using RNeasy Mini kit (Qiagen). cDNA was synthesized using oligo-dT primer and MMLV reverse transcriptase (SuperScript III, Invitrogen) according to the manufacture's instruction. Two micrograms of total RNAs was used for cDNA synthesis. For multiplex RT-PCR, we have tested individual primer sets for their potential crosstalk with others and removed or replaced them if they were found to affect the amplification of the other target sequences in the reaction. We finally selected 13 pluripotency-related gene sequences and 20 reprogramming-related sequences for multiplexing, each primer set of which displays relatively independent PCR amplification in the PCR reaction. With a whole single colony as a template, first-round PCR was conducted using one-step RT-PCR kit (Qiagen, USA) as described in the supplier's manual. RT-PCR and subsequent 1st PCR were done with the same sets of primers (for primer information, see Supplementary Table S1 (in the Supplementary Material available online at http://dx.doi.org/10.1155/2015/195928) for pluripotency genes and Table S2 for reprogramming-related genes). RT-PCR was done at 50°C for 30 min in a thermal cycler (DNA engine, BioRad, USA) and then 1st PCR was done in the resultant RT-PCR mixture; the cycles consisted of 15 cycles of 94°C for 30 s, 56°C for 30 s, and 72°C for 30 s. For a second-round PCR, the product from the 1st PCR was diluted hundredth and used as template in the second-round PCR with nested sets of primers (see Tables S1 and S2). We used a hot-start-based multiplex PCR kit (Solgent, Korea) for the second-round PCR, with the same cycling condition as in the 1st PCR but for 25 cycles. The 2nd PCR products were resolved on 8% PAGE gel. Band density of each PCR amplicon was measured using TINA2.0 or AxioVision intensity profiling tool (v4.8) both of which revealed very similar results.

### 2.5. Quantitative Measurement of Gene Expression Profile

Variation of a colony's GEP from the reference GEP was measured using the term wRMSD. We considered each gene expression to be an independent event; therefore, we combined all of the expression measurements of each sample in the calculation of the wRMSD. In order to minimize the bias that could result from a measurement error of a GEP with a low coefficient of variation (CV), the deviation of each gene expression level from the mean was weighted with the CV of the gene in the group. The wRMSD was obtained with

(1) $$\begin{array}{c} \text{wRMSD}=\sqrt{\sum w_{i}\cdot {\left({Em}_{i}-E_{i}\right)}^{2}}, \end{array}$$

where *w*_*i*_, *Em*_*i*_, and *E*_*i*_ are the values obtained from the *i*th gene of interest for the weight of the mean square deviation of the gene expression, the reference expression level (e.g., the mean expression level in the group), and the expression level of the gene, respectively. The weight (*w*_*i*_) is the proportion of the coefficient of variation (CV) for the expression level of the *i*th gene to the sum of CV for those of all genes in the group and was obtained with

(2) $$\begin{array}{c} w_{i}=\frac{{\text{CV}}_{i}}{\sum {\text{CV}}_{i}}. \end{array}$$

## 3. Results

### 3.1. Characterization of Initially Formed-iPSC Colonies Derived from NGFP and 4F2A Reprogrammable Cells

**Figure 1:**
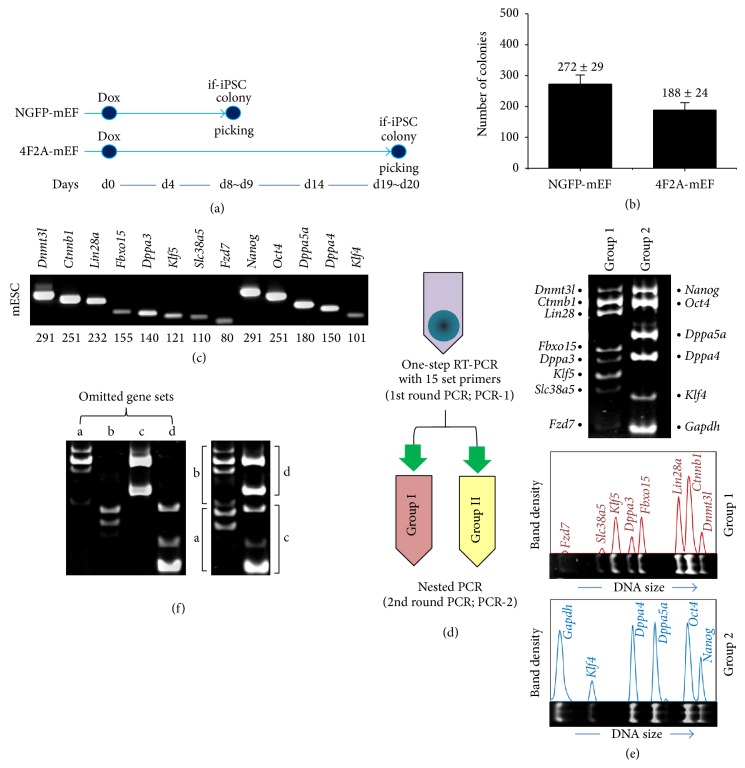
Multiplex reverse transcription-polymerase chain reaction for pluripotency genes in NGFP and 4F2A cells. (a) A schedule for doxycycline (Dox) induction in NGFP-mEF and 4F2A-mEF culture. Initially formed-iPSC (if-iPSC) colonies of proper sizes were picked at indicated times. Dox treatment was maintained until colony picking. (b) The number of pre-iPSC colonies emerged from NGFP-mEF (272 ± 29 per 2 × 10^4^ cells) and 4F2A-mEF (188 ± 24). Mean ± standard deviation. *n* = 12. (c) RT-PCR of individual genes using cDNA from mouse J1 mESCs. The size of PCR amplicon is indicated below. (d) Multiplex RT-PCR strategy. One-step RT-PCR was used as the first round of PCR (PCR-1) and the products advanced to the second-round, nested PCR (PCR-2). (e) A representative gel image (upper) and the corresponding band-intensity profiles of the groups 1 and 2 genes (lower). (f) Control experiments for multiplex RT-PCR using total RNA from J1 mESCs. Omitting each set of primers indicated (a–d) in the PCR-1 cocktail does not greatly change the overall pattern and quantity of the PCR-2 products of the remaining genes in each group, indicating no severe interference between the multiple sets of primers during multiplex RT-PCR.

We injected stabilized NGFP-iPSCs [10, 24] into mouse blastocysts and from the resulting chimeric fetuses we obtained embryonic fibroblasts (NGFP-mEFs). 4F2A-mEFs were obtained from fetuses of the 4F2A transgenic mice [12]. Both NGFP-mEF and 4F2A-mEF were cultured in the presence of Dox to induce iPSCs. Initial colonies began to form at different schedules, so the 4F2A if-iPSC colonies were delayed to form ten more days (Figure 1(a)). Alkaline phosphatase (AP) staining showed that the numbers of if-iPSC colonies generated were highly constant between replications, fewer in the 4F2A-mEF culture (188 ± 24; mean ± standard deviation) than in the NGFP-mEF culture (272 ± 29; *P* < 0.001; Figure 1(b)). The NGFP and 4F2A if-iPSC colonies were individually collected from each culture at 8-9 days of induction and at 19-20 days, respectively. We selected if-iPSC and mESC colonies of similar sizes only; the average number of cells in single if-iPSC colony was 2571 ± 160, slightly less than that (2825 ± 267) of mESC colony, probably reflecting the larger size of if-iPSCs.

### 3.2. Generating GEPs of Pluripotency-Related Genes Using Multiplex RT-PCR

In order to investigate expression signature of pluripotency genes in individual NGFP and 4F2A if-iPSC colonies, we chose a group of genes that are highly expressed in mESCs [23, 25] and identified their expression in mESCs by RT-PCR (Figure 1(c)). For multiplex RT-PCR for these genes, we used a 2-step PCR strategy as before [26]; the RT-PCR product from the first round (PCR-1) was diluted and used as the template in the second round of PCR (nested PCR; PCR-2; Figure 1(d)). The 14 genes used for expression profiling, including* Gapdh* as an internal standard, were divided into groups 1 and 2. In Figure 1(e), a representative result of multiplex RT-PCR using a single mESC colony as template is shown, together with the band density profile of the amplicons. When primer sets corresponding to 3-4 genes were omitted from PCR-1 cocktail, the amounts of PCR-2 products of the remaining genes were not quite changed (a–d in Figure 1(f)). This indicates that there is no severe cross-interference between the multiple sets of primers in the multiplex RT-PCR.

**Figure 2:**
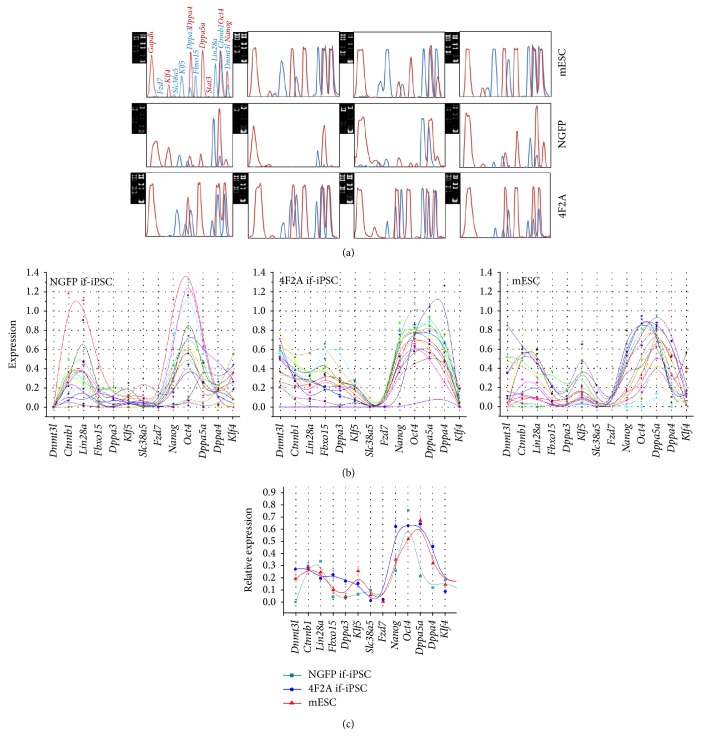
Expression profiles of pluripotency genes in single if-iPSC colonies from NGFP, 4F2A, and mESC groups. (a) Representative gene expression density profiles of single if-iPSC colonies in the mESC, NGFP if-iPSC, and 4F2A if-iPSC groups. Each panel represents a density profile of a single colony (4 profiles per each group) and the corresponding gel image is shown left with lanes of individual gene groups (Figure 1(e)). AxioVision intensity profile tool (v4.8) was used for peak profiles. (b) Gene expression profiles (GEPs) of individual colonies. Spline-type plots are shown where individual lines in different colors denote the expression profiles of the pluripotency genes in individual colonies. (c) Mean GEPs of individual iPSC colony groups. The* Gapdh* level is set as the normalization standard.* Slc38a5* is included as a control that was previously shown to be more expressed in if-iPSCs than in stabilized iPSC and ESCs [23]. Error bars, standard error.

We first examined gene expression in mEFs (~3,000 cells). Multiplex RT-PCR showed that both NGFP-mEFs and 4F2A-mEFs express* Klf4 *(Supplementary Figure S1A). Differing from the 4F2A-mEFs, interestingly, NGFP-mEFs expressed several genes, including* Ctnnb1*,* Lin28a*,* Klf5*, and* Oct4*, though at a very low level compared with level of* Gapdh *(Figure S1B). Next, multiplex RT-PCR was performed with single colonies, mESC (*n* = 27), NGFP if-iPSC (*n* = 27), and 4F2A if-iPSC colonies (*n* = 28).  Figure 2(a) shows representative band-intensity profiles of individual colonies (see Supplementary Figure S2 for the rest). To determine gene expression heterogeneity among individual colonies of each iPSC group, GEPs of single colonies were plotted into a spline type (Figure 2(b)). Individual colonies had their own GEPs for the pluripotency genes that could be used to identify these colonies but, nevertheless, they formed a distinctive pattern with some degree of uniformity in each group, different from the patterns of the other two groups. The result indicates that NGFP and 4F2A if-iPSC groups possess unique GEPs differentiated from mESCs' standard GEPs.  Figure 2(c) shows the mean GEP of each group that can be considered as an expression signature of each group; notably, overall expression levels of 4F2A if-iPSCs were shown to be higher than NGFP if-iPSCs. Using a quantitative real-time PCR, we confirmed the gene expression differences among the colony groups (Supplementary Figure S3).

### 3.3. Calculating Difference in GEPs of Pluripotency Genes between 4F2A if-iPSCs and NGFP if-iPSCs

**Figure 3:**
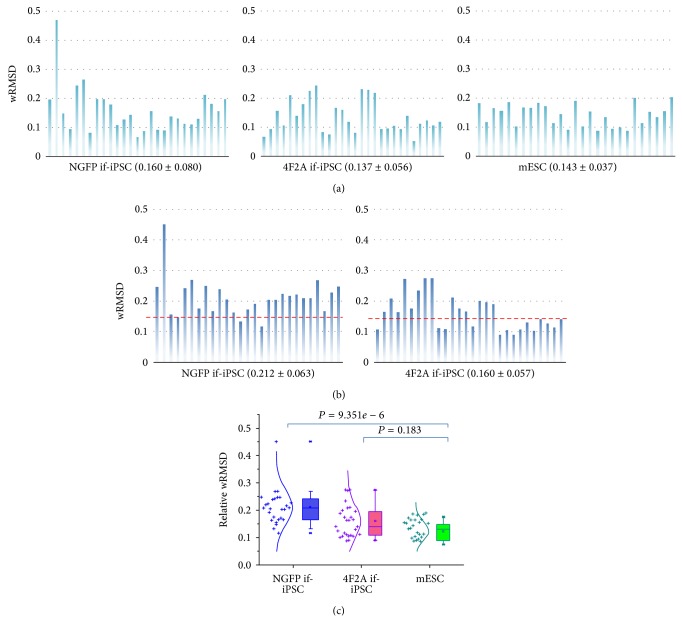
wRMSD distributions of NGFP if-iPSC and 4F2A if-iPSC colonies relative to those of mESC colonies. (a) The distribution of weighted root mean square deviation (wRMSD) of individual colonies in each group. The deviation of each if-iPSC colony's GEP from the mean GEP of each group was estimated using wRMSD. Numbers in parentheses denote mean ± standard deviation. The closer the wRMSD is to zero, the closer the GEP is to the mean GEP of its own group. (b) Relative wRMSD obtained by calculating the deviations of the gene expression in each NGFP if-iPSC and 4F2A if-iPSC colony from the mean GEP of the mESC group. Dashed red line in each panel indicates the mean wRMSD (0.143 ± 0.037) of the mESC group as the reference. (c) Statistical difference between the groups. The *P* values between different colony groups are indicated above (*t*-test). The wRMSDs of individual colonies are shown as crosses together with a normal distribution curve on the left of each box plot. Box whiskers indicate the range from 5% to 95%.

To assess how different the GEP of each colony was from those of the others within the same group, the deviation of each colony's GEP from the mean GEP of each group was estimated using a weighted root mean square deviation (wRMSD) [26]. When the GEPs of representative colonies with the lowest and highest wRMSDs obtained from statistical calculation were shown along with the mean GEP in each group, it was clear that the lower the value of the wRMSD is, the closer the GEP of a colony is to the mean GEP (Supplementary Figure S4).  Figure 3(a) represents the distribution of wRMSDs in each group; similar mean wRMSD values among the groups illustrate similar levels of uniformity in the gene expression patterns among them.

We then measured the difference in the GEPs of NGFP if-iPSC and 4F2A if-iPSC colonies compared to the mESC colonies, which was obtained by calculating the deviations of the GEPs of each colony in the NGFP and 4F2A if-iPSC groups from the mean GEP of the mESC group. As in Figure 3(b), which shows the distribution of relative wRMSD values, the mean wRMSD value of NGFP if-iPSC colonies was shown to be higher than that of 4F2A if-iPSC colonies. It indicates that NGFP if-iPSC colonies are more different from mESCs than 4F2A if-iPSC colonies. Statistics with wRMSD values of individual colonies' GEPs revealed that the NGFP if-iPSCs were significantly different from the mESC group (*P* = 9.351*e* − 6; *t*-test) but, interestingly, no significance level was observed between the mESC and the 4F2A if-iPSC group (*P* = 0.183; Figure 3(c)).

### 3.4. High Frequency of Endogenous* Nanog* and* Sox2* Gene Expressions in 4F2A if-iPSCs

**Figure 4:**
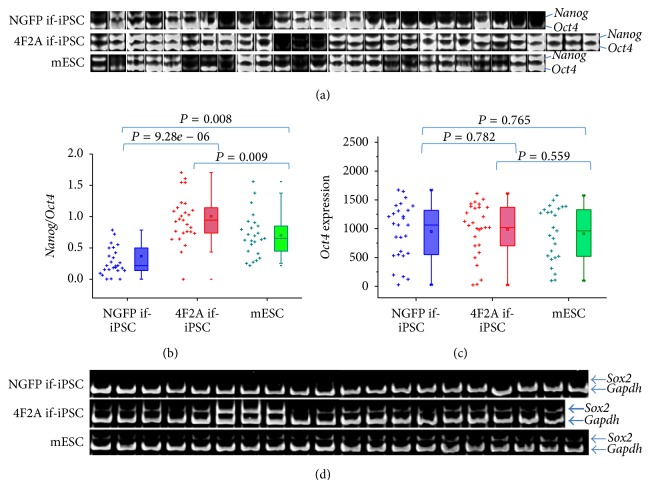
Higher frequency of derepression of* Nanog* and* Sox2* genes in 4F2A if-iPSC colonies than NGFP if-iPSC colonies. (a)–(c) Expression levels of* Nanog *to* Oct4 *genes in NGF if-iPSC, 4F2A if-iPSC, and mESC colonies. Expressions of* Nanog* (upper band) and* Oct4* (lower) in individual if-iPSC colonies are presented in gel images (a) and by statistics using a box-scatter plot (b).* Oct4* expression levels are shown in (c). In (a) and (b) the expression levels of* Nanog* and* Oct4* are of their own, not normalized with* Gapdh*. The values of individual colonies are shown as dots on the left of each box plot. The mean values are indicated in the boxes (circles). Box whiskers indicate the range from 5% to 95%. (d) Duplex RT-PCR showing expression levels of* Sox2* relative to* Gapdh* in single colonies. Expression frequency is higher in 4F2A if-iPSC colonies than in NGFP if-iPSC colonies. The primer sets used were designed to specifically recognize 3′-untranslated regions (UTR) of endogenous* Oct4*,* Nanog*, and* Sox2* genes which were absent in NGFP and 4F2A reprogramming cassettes.* Gapdh*, internal control.

Expression levels were overall higher in 4F2A if-iPSC colonies than those in NGFP if-iPSC colonies and in particular the levels of* Dnmt3l*,* Fbxo15*,* Dppa3*,* Nanog*,* Dppa5a*, and* Dppa4* genes were significantly higher in 4F2A if-iPSCs (*P* < 0.001; Figure 2(c)).* Nanog* was particularly interesting because of its variable expression level among different colonies and groups (Figure 4(a)). The levels of endogenous* Nanog* relative to endogenous* Oct4* in individual colonies were markedly low in the NGFP if-iPSC group (0.369 ± 0.509) compared with the 4F2A if-iPSC group (1.005 ± 0.451), displaying a significant difference between them (*P* = 9.28*e* − 6; Figure 4(b)). In contrast,* Oct4* levels were relatively similar in these three groups (Figure 4(c)). When we additionally examined expression levels of endogenous* Sox2 *in if-iPSC colonies from a separate experiment,* Sox2* expression was little detected in NGFP if-iPSC colonies and the levels were again shown to be coherently higher in 4F2A if-iPSC group than NGFP if-iPSC (Figure 4(d)). The result demonstrates that the higher proportion of 4F2A if-iPSC colonies proceeds to derepress their endogenous stemness genes.

### 3.5. wRMSD Analysis of GEPs of Reprogramming-Related Genes between 4F2A if-iPSCs and NGFP if-iPSCs

**Figure 5:**
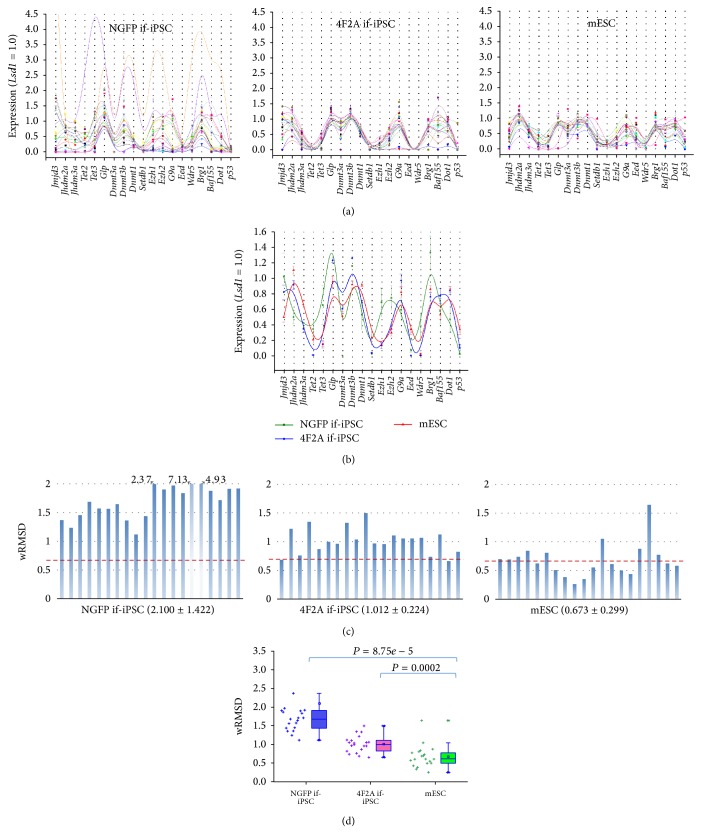
wRMSD analysis of gene expression profiles of reprogramming-related genes between 4F2A if-iPSCs and NGFP if-iPSCs. (a) GEPs of reprogramming-related genes in individual colonies. Spline-type plots are shown where individual lines in different colors denote the expression profiles of reprogramming-related genes in individual colonies. (b) Mean GEPs of individual colony groups. Error bars, standard error. (c) Relative wRMSD. It is obtained by assessing the deviations of the gene expression in each NGFP if-iPSC and 4F2A if-iPSC colony from the mean GEP of the mESC group. A reference line in red on each panel denotes the mean wRMSD in the mESC colony group. (d) Statistical difference between the groups. The *P* values are indicated above (*t*-test). The wRMSDs of individual colonies are shown as crosses on the left of each box plot. The wRMSD range is set to 0.0–2.0 for a group-to-group comparison, and those wRMSDs beyond 2.0 are denoted above. The mean values are indicated in the boxes (circles). Box whiskers indicate the range from 5% to 95%.

The choice of target genes could possibly affect sensitivity of the incoming results. So, we examined another set of genes which we arbitrarily selected from the list of genes that are well known implicated in reprogramming [19, 24] (Supplementary Figure S5). In total, twenty genes were examined in individual if-iPSC colonies by multiplex RT-PCR (Supplementary Figure S6). GEPs of the genes in individual colonies are shown in Figure 5(a) and the mean GEPs for the three groups are shown in Figure 5(b). NGFP if-iPSCs' GEPs revealed a large variance whereas 4F2A if-iPSCs' GEPs looked even steadier than the mESCs' GEPs. Unlike the pluripotency genes (Figure 2(c)) that were more expressed in 4F2A if-iPSC colonies than NGFP if-iPSC colonies, these reprogramming-related genes tended, oppositely, to be more expressed in NGFP if-iPSC. wRMSD analysis indicates that the NGFP if-iPSCs' GEPs were more deviated from the mESC standard than the 4F2A if-iPSCs' GEPs (Figure 5(c)). This conforms to the result from the analysis of stemness-related genes (Figures 3(b) and 3(c)). Statistics in Figure 5(d) represents the relative distance between the colony groups, with the 4F2A if-iPSCs closer to mESCs, the same as in the statistics of wRMSDs of the GEPs of pluripotency genes (Figure 3(c)). Notably, whereas the GEP difference in pluripotency genes was not different between 4F2A if-iPSCs and mESCs (*P* = 0.183), the GEP difference in reprogramming-related genes was significantly different between them (*P* = 0.00024). In conclusion, notwithstanding their indistinguishable colony morphology, the 4F2A if-iPSCs and the NGFP if-iPSCs have quite different GEPs and biochemical nature.

## 4. Discussion

Initial NGFP cell clumps begin to appear in about 4-5 days after Dox exposure, while 4F2A counterparts do so in about 15 days. This difference in the time table surely originates from the different transgenic composition of the two mEFs. Both types of cells are derived from the same mESC clone containing a reverse tetracycline transactivator (*rtTA*) gene targeted to the ROSA26 locus (*ROSA26-M2rtTA*, [15]). mEFs obtained from the* ROSA26-M2rtTA* fetus, which also carried* GFP* targeted to the endogenous* Nanog* locus, were infected with four lentiviruses to make the primary NGFP-iPSCs [10]; the resulting iPSC lines contain multiple (>7) proviruses for the four transcription factors [12]. The secondary NGFP-mEFs we here used were obtained from E13.5 fetuses derived by blastocyst injection of the primary NGFP-iPSCs. In case of the 4F2A-mEFs, on the other hand, a single polycistronic cassette (*4F2A*; Oct4-Sox2-Klf4-c-Myc) was inserted into the 3′-untranslated region of* Col1a1* locus in the* ROSA26-M2rtTA* mESC [12]. The 4F2A-mEFs were obtained from E13.5 transgenic fetuses produced by intercrossing the resulting transgenic mice. The multicopy transgenes in the NGFP-mEFs likely achieve the higher expression of the four factors than the single-copy transgene in 4F2A-mEFs, which possibly forces NGFP-mEFs to quickly form plenty more of mESC-like colonies than 4F2A-mEFs do.

Nanog is a pluripotency factor, and deriving ESCs and iPSCs requires normal Nanog function [17]. Furthermore, Nanog enhances the transfer of pluripotency by cell fusion [27] and facilitates direct reprogramming of human cells [5, 28–30]; in addition, its overexpression together with overexpression of the four factors has been shown to enhance reprogramming [17, 24, 31]. Therefore, the derepression of the endogenous* Nanog* locus by reprogramming factors must be an important requirement for activation of endogenous pluripotency factors [19]. The 4F2A if-iPSCs expressed higher* Nanog* levels than the NGFP if-iPSCs (Figure 4(a)), which may indicate that reprogramming events are occurring more efficiently in the 4F2A if-iPSCs. Agreeing with this, when endogenous* Sox2* gene was examined, it was more frequently derepressed in 4F2A if-iPSC group (Figure 4(d)). The higher expressions of endogenous* Nanog* and* Sox2* genes would provide positive feedback to the cells spurting further reprogramming. Notably, the expression levels of endogenous stemness genes in 4F2A if-iPSCs appear to overshoot, as the endogenous* Nanog* and* Sox2* levels in them were even higher than those of mESCs. This may be related with the prolonged schedule of Dox treatment in 4F2A cells (Figure 1(a)). This overflowing level could be only transient and help the cells pass the crest of reprogramming. However, in case of the overexpression lasting irrelevantly longer, there is a concern that the overexpression of the stemness genes could interfere with cellular reprogramming progressing to the next step.

The results indicate that although 4F2A and the NGFP cells form colonies of indistinguishable morphology, these two types of colonies have their own distinctive GEPs and different biochemical nature in them. We have no idea whether 4F2A if-iPSCs and NGFP if-iPSCs have gone through a similar amount in total of exogenous activity of the reprogramming factors, but what makes difference between these cells is most likely present in their modes of action of the transcription factors, the short-but-full mode in NGFP if-iPSCs* versus* the long-but-thin mode in 4F2A if-iPSCs. Choosing the better for reprogramming, our results favor the latter by their more closeness in GEP to the regular mESCs.

Of the analytical methods researchers have adopted test difference or variation in gene expression between samples; Principal Component Analysis (PCA) is a popular one extensively used to simplify rather complex results such as large-scale gene expression data for a preliminary observation [32]. Through PCA test, correlation between samples can be “graphically” displayed in a simple single plot. However, the math behind PCA is not simple enough for researchers to easily go through and, without thorough understanding of the math, it is difficult to assess variation between samples “numerically.” Besides, to run the statistical test like PCA, a certain level of computer programming knowledge is also required. wRMSD is, in contrast, a very simple way to test efficiency of iPSC techniques or protocols by comparing expression of genes and is a method highly accessible to researchers who are familiar with spreadsheet software. In addition, wRMSD analysis gives rise to detailed variations of individual variables (or genes) as well as variation overview of whole sample, which are the types of results many dimension-reduction methods including PCA are unable to produce. Therefore, the wRMSD would be the better choice especially in case that the number of target genes to be analyzed is limited.

## Supplementary Material

Supplementary Table S1. Primer sequence information (pluripotency genes).
Supplementary Table S2. Primer sequence information (reprogramming-related genes).
Supplementary Fig. S1. Expression levels of pluripotency genes in NGFP-mEFs and 4F2A-mEFs.
Supplementary Fig. S2. Density profiles of gene expressions in single pre-iPSC colonies in NGFP, 4F2A, and mESC groups.
Supplementary Fig. S3. A quantitative real-time PCR. Complementary DNAs obtained from pooled colonies (20 per group) were analyzed for Nanog, Dppa5a, and Dppa4.
Supplementary Fig. S4. Gene expression profiles (GEPs) of representative pre-iPSC colonies with different wRMSDs.
Supplementary Fig. S5. Single-gene RT-PCR using cDNA from J1 mESCs.
Supplementary Fig. S6. Multiplex RT-PCR for reprogramming-related genes using single pre-iPSC colonies.

## Abbreviations

if-iPSC: Initially formed-induced pluripotent stem cells
wRMSD: Weighted root mean square deviation
GEP: Gene expression profile
Dox: Doxycycline
mEF: Mouse embryonic fibroblast.
