# Supplementary material for: Differences in the Gene Expression Profiles of Slow- and Fast-Forming Preinduced Pluripotent Stem Cell Colonies
Source: Stem Cells Int. 2015 Apr 7;2015:195928. doi: 10.1155/2015/195928 (PMC4405011; doi:10.1155/2015/195928)
Supplement: Supplementary file 1 — Supplementary Table S1. Primer sequence information (pluripotency genes). Supplementary Table S2. Primer sequence information (reprogramming-related genes). Supplementary Fig. S1. Expression levels of pluripotency genes in NGFP-mEFs and 4F2A-mEFs. Supplementary Fig. S2. Density profiles of gene expressions in single pre-iPSC colonies in NGFP, 4F2A, and mESC groups. Supplementary Fig. S3. A quantitative real-time PCR. Complementary DNAs obtained from pooled colonies (20 per group) were analyzed for Nanog, Dppa5a, and Dppa4. Supplementary Fig. S4. Gene expression profiles (GEPs) of representative pre-iPSC colonies with different wRMSDs. Supplementary Fig. S5. Single-gene RT-PCR using cDNA from J1 mESCs. Supplementary Fig. S6. Multiplex RT-PCR for reprogramming-related genes using single pre-iPSC colonies. [file 195928.f1.pdf]

Supplementary Table S1. Primer sequence information (pluripotency genes)

Gene	Primer sequence<br>1st round PCR	 2nd round PCR	Accession# (mouse)
Dnmt3l	ATGATGGACACCAGAGTTAC<br>AAAAGCCCAAACTCTTTAGT	TGCTGTTCCGGGGGTACCCT<br>AGGCTCAGTACCCGCACTGG	NM_019448.3
Ctnnb1	CATTAATAGCAGCCTTTCTC<br>CTACACCATTACTCGGTTCT	AACCAAGCTGAGTTTCCTAT<br>AGAACATAACACTACTTAAA	NM_007614.3
Lin28a	AAATTTATGAAGACGAGGTG<br>TGTGTTCTATTGCATTTGTC	GGAATCTTGGGCTTCATTTGT<br>TTAGCTATCAACCATCACGCT	NM_145833.1
Fbxo15	AATTTACGCAAGTCTGCTAT<br>ATCTCCATAAAAGGTCATTG	TCTGTGTAAATCCTGGCCTC<br>CACAAATGTAGTGTCGGGAG	NM_015798.3
Dppa3	AAGATGAGAAGACTTGTTCG<br>AAATAGCTTTCACATCTGCT	TTGAGCAGAGACAAAAAAGG<br>TGTAAGCTCCTAATTCTTCC	NM_139218.1
Klf5	GATAGACAAGCTGAGATGCT<br>TATAAACTTTTGTGCAACCA	CCGCTACATTGCTTCCAAAC<br>TTCTCCAGATCCGGGTTACT	NM_009769.4
Slc38a5	CACACTGGAGTCATCTTCTT<br>AACAGGTAACTGGACATAGC	ATTGCTCTCCTGTCTTCTTA<br>TTCCCGGCAGGTCCAAATGC	NM_172479.2
Fzd7	TGGTTGAGATAAACATTCCT<br>AAAACAAAAACAGCAAAAAT	TACCCATTCAGGCACACGTT<br>TGAATTAAATCTGTTTAAAACAAATA	NM_008057.3
Nanog	CTAGCAATGGTCTGATTCAGAAG<br>TAGTGGCTTCCAAATTCACCTCC	CACCAGTGGAGTATCCCAGCAT<br>TGGCAGAGAAGTTTTGCTGCAAC	NM_028016.2
Oct4	CCCAGGGGGGGCTGTATCCTT<br>CACTGAGCTTCTTTCCCCATCC	CCCAGGTCCCCACTTTGGCAC<br>ACCCCTGTTGTGCTTTTAATCCC	NM_001252452.1
Dppa5a	CTGAAGAACCTGGAATCTC<br>ATTTAGAGTGGTAACCTTGG	GAAGAACTTATCGAGGTCTT<br>GACTGGAAACTGGCTTCACT	NM_025274.3
Dppa4	AGTGCCAGCCGATGCTTCGT<br>GATTTCTATCAGTCTCAGCGT	TCCTGGGAACTCGCGTGGTT<br>TTGGGGCACAGCATGTTGTC	NM_028610.2
Klf4	AGTTTTGTCTTCCGATCTACATTG<br>TACATATTTATTAAGGCAAGGAAC	GGTTTATTTCTGTAACATTTTTATG<br>TTAAAGGCATACTTGGGAATAAAC	NM_010637.3
Gapdh	GACCTCAACTACATGGTCTACAT<br>GAGTGAGTTGTCATATTTCTCGT	TGGAGAAGGCCGGGGCCCAC<br>AAACATGGGGGCATCGGCAG	NM_008084.2

Supplementary Table S2. Primer sequence information (reprogramming-related genes)

Gene	Primer sequence<br>1st round PCR	 2nd round PCR	Accession#
Jmjd3	CTCACCATCAGCCACTGTG<br>AGCTCTCACAGGGCCAGAT	TGCCAGCAAGAATGCCAAG<br>CCAGAGTCTTGGTGGAGAAA	NM_001017426.1
Jhdm2a	AGCATTCTTGGCTTTGACA<br>CATCAGATCATCAAACCTG	GGCAGCCAGTGATGGTGT<br>CCTTAAGTTTCAACACCATTG	NM_001038695.2
Jhdm3a	TTCTACCAGTGTGAGGTG<br>TCTCATTGAAGCGCATGTC	CATAGTGAGTCAGGACTGTC<br>AGTCTAGACTTGACTCTCTT	NM_001161823.1
Tet2	GGAGGAAGGCAGTGACATC<br>ATAAACTGCATTCATAACA	CGATACAAGTGCTTCTGCT<br>AAAAAAACCCTAACAAGTT	NM_001040400.2
Tet3	GGCATGGCCTACACTTCAT<br>GGCTCGGCTGCCACTGTGT	TTCTCTCCTCCCTCTCCCT<br>GTTCTCATTTTCAAAGAGC	NM_183138.2
Glp	TGGTCAAGTATGAGCTGATGC<br>TGCTATGGTCACCTCTTTG	GCGCCCATCCAACAAAGCA<br>CCTTGGAAGCTTCTTCCCC	NM_001012518.2
Dnmt3a	TCAAGGAGATCATTGATG<br>TGTTCCCACACATGAGCA	GCGCACAAGGGAGCGGCTG<br>AGATGGTGCAATAGGACTG	NM_007872.4<br>NM_001271753.1
Dnmt3b	CCTACCCGGAATGAACAG<br>ACAGGCAAAGTAGTCCTTC	AGCTCGAGCTGCAGGACT<br>AGATCCTTTCGAGCTCAGTG	NM_001003960.3
Lsd1	TCTGGCTTGGCAGCAGCTC<br>GTGGCTTCTAGCAACCGGT	ACTTCTGGAAGCCAGGGATC<br>CTTGATCTTGGCCAGTTCCA	NM_133872.2
Dnmt1	CAGTGGCATGAACCGCTTCA<br>GTCTGGGCCACGCCATACT	GCTACTGTGACTACTACCG<br>AAAGGTGCACTGGTAGCC	NM_010066.4
Setdb1	CACACGCCAGTTCTATGA<br>TCAGGAAGACCCAGTTCCT	GGCCGCTACCTCAATCAC<br>CCGGATTCTCTTGCTGGC	NM_001163641.1
Ezh1	TGCGGCTATGCCTGGTTGG<br>GTTTTTGTGTCAAGTCAGC	GCTTTCTTCAGGGTAGCTG<br>TCCTCTTCCTCCTTCAGAA	NM_007970.2
Ezh2	CCGCAGAAGAGGAAGACTT<br>GTTTCCTTTTCTTCTTTCT	ACCATCAGTGTGCTGGAGT<br>CCAATGAGGACTCTAAACA	NM_007971.2
G9a	GACAGAACGCGGGTTTGAG<br>CAGGCCTTATGGAAGCGG	CCGCATGGAGGCTCCCAAG<br>ATGCGGGCTCGATGGGCCT	NM_145830.1
Eed	CTGAGTGCTGATTATGATC<br>TATGAGGATCTTCTACTTC	TTCCTGACTTTTCTACCAG<br>ACTTGGAAAGTATCAAATC	NM_021876.3
Wdr5	TTTTTCTCTGTGGTTGTTG<br>CCAGAGACCTTTCCAACTC	GAACAGGACCTACAGCCCT<br>TCCATACCCACCTTTTATT	NM_080848.2
Brg1	AGGAGTACCTCAACAGCAT<br>TGCCTTCTTCTTTTTCTTT	TCACAGATCAGTCACAGGC<br>TGTCTGCTGCAGAAGGTAG	NM_001174078.1
Baf155	GCCCGGCAGCAAATGGAGC<br>GGCTAGGGAGTGGCCGAGG	GCACCATCAGATGCCGCCA<br>GATGTTTCCGGGCATTGGA	NM_009211.2
Dot1	GTGGACAGTCCGCTACAGG<br>TTGCGTTGCCCTCACCCTT	GGGGGCCTTGCTGCAGACC<br>TTGGCCTCTGTGGTACTGT	NM_199322.1
p53	TTGCGTTGCCCTCACCCTT<br>ACAACTGGCTGGATAGAAT	AGACCAAGAAGGGCCAGTC<br>GGCTGGTGATGGGGACGGG	NM_011640.3

Supplementary Figure legends

Fig. S1. Expression levels of pluripotency genes in NGFP-mEFs and 4F2A-mEFs. **A**, Multiplex RT-PCR results. Each RT-PCR set-up employed about 3,000 cells as a template, which were repeated four times. Wherever PCR bands are present are denoted by gene symbols with purple dots. B, Expression levels of pluripotency genes. Error bars, the standard deviation of the mean (STD). *Gapdh* level = 1.0

Fig. S2. Density profiles of gene expressions in single pre-iPSC colonies in NGFP, 4F2A, and mESC groups. Insets, corresponding gel images.

Fig. S3. A quantitative real-time PCR. Complementary DNAs obtained from pooled colonies (20 per group) were analyzed for *Nanog, Dppa5a*, and *Dppa4*. One-way ANOVA and the Bonferroni post hoc test were used for statistical analysis. Asterisks (p < 0.001) denote a significant difference between samples. Error bars, standard deviation.

Fig. S4. Gene expression profiles (GEPs) of representative pre-iPSC colonies with the lowest (minimal weighted root mean square deviation, [min wRMSD] in green) and the highest wRMSD (max wRMSD in purple) are shown along with the mean GEP (in red) for each group.

Fig. S5. Single-gene RT-PCR using cDNA from J1 mESCs. The size of PCR amplicon for the indicated reprogramming-related genes is indicated below.

Fig. S6. Multiplex RT-PCR for reprogramming-related genes using single pre-iPSC colonies. Above, a representative gel image (left) and the relative PCR band

positions of the genes analyzed (right). Below, gel images of multiplex RT-PCR products. DNA markers are also shown in each gel.

Figure S1

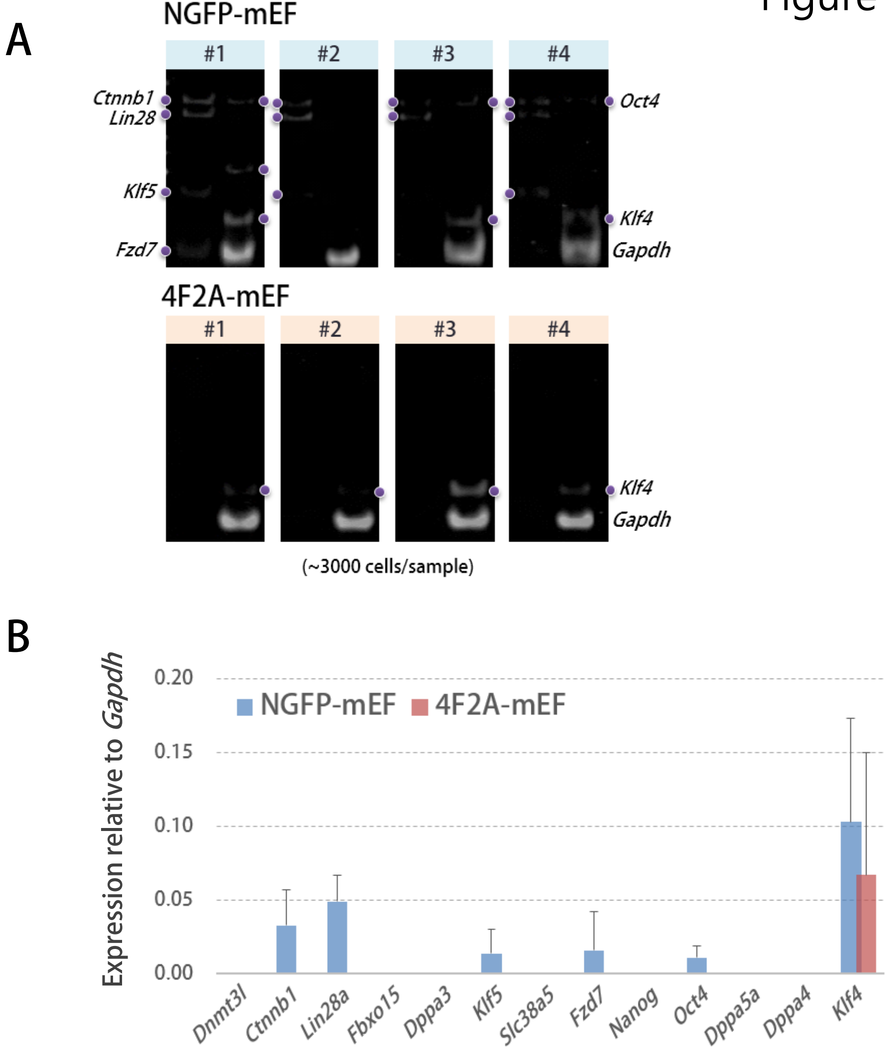

Fig. S1. Expression levels of pluripotency genes in NGFP-mEFs and 4F2A-mEFs.

Figure S2

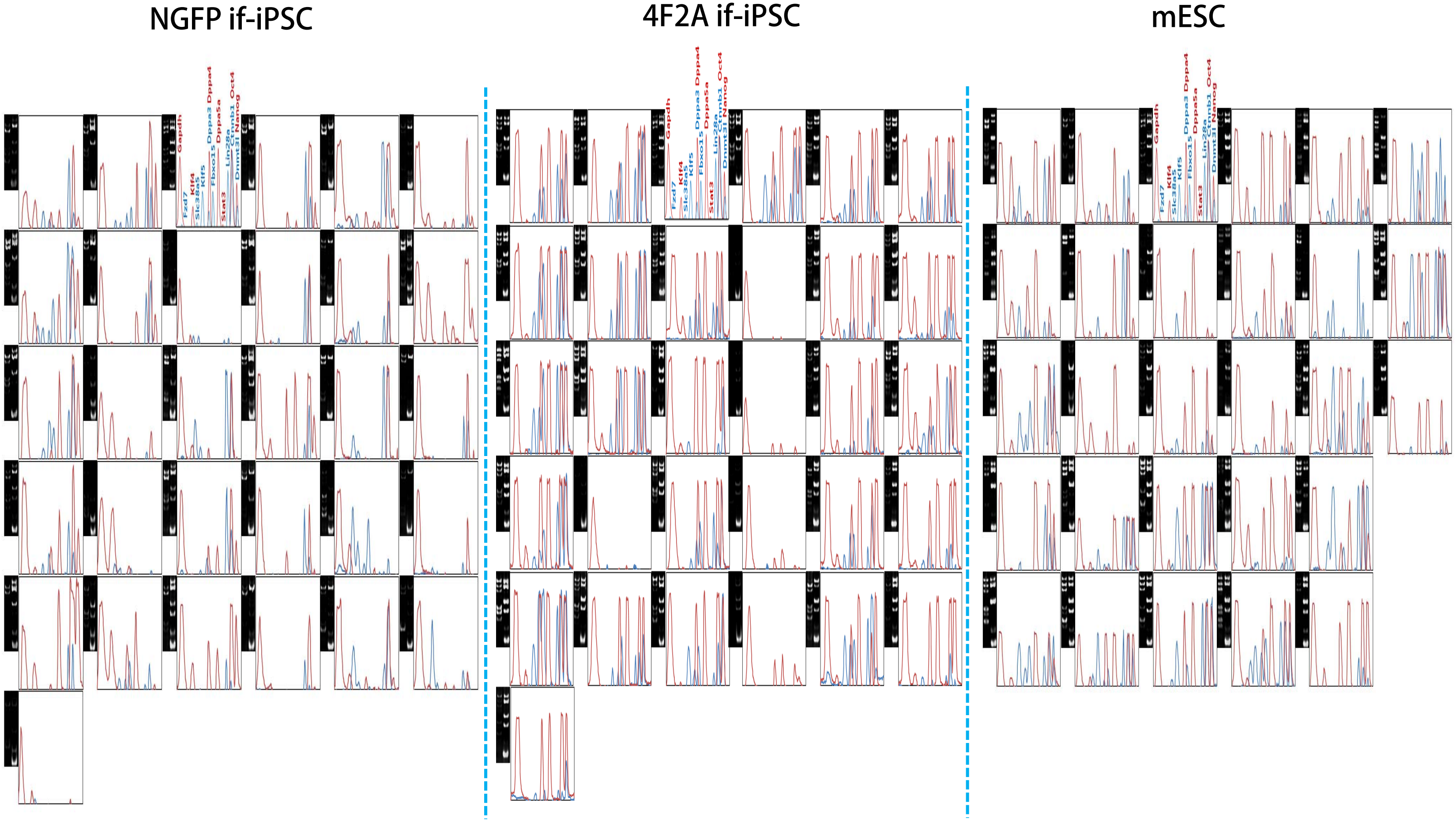

Fig. S2. Density profiles of gene expressions in single if-iPSC colonies

Figure S3

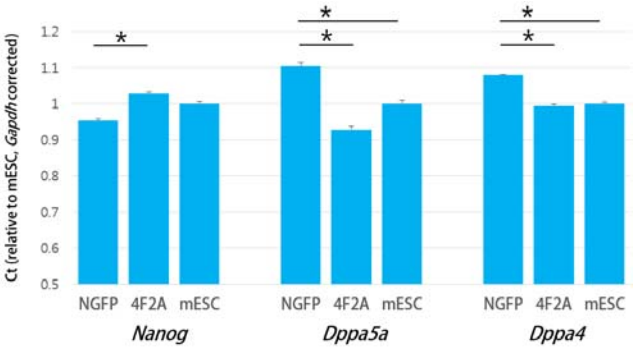

Fig. S3. A quantitative real-time PCR.

Figure S4

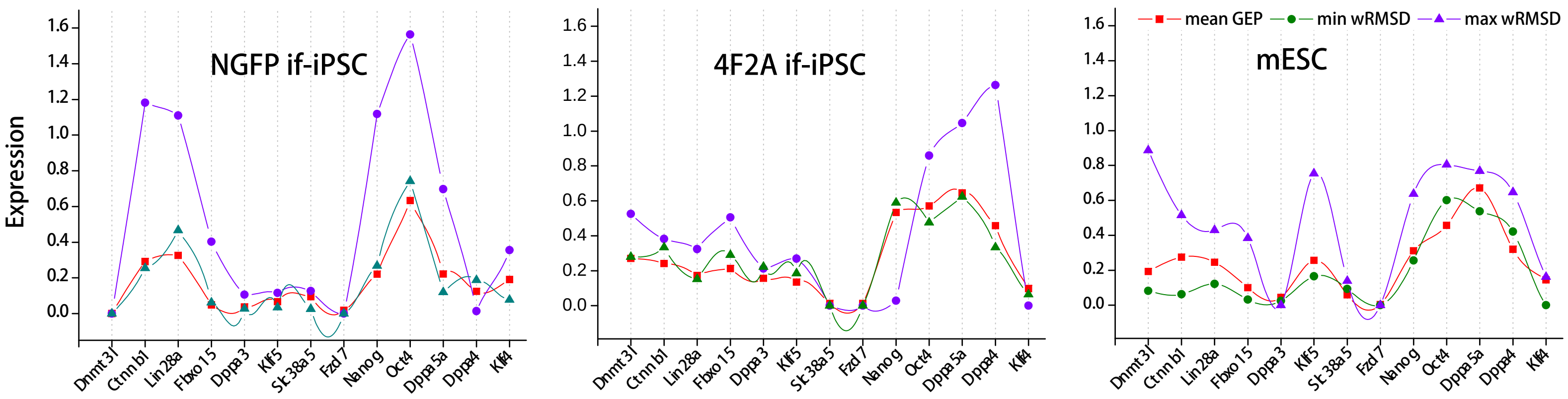

Fig. S3. Gene expression profiles (GEPs) of representative if-iPSC colonies with different wRMSDs

Figure S5

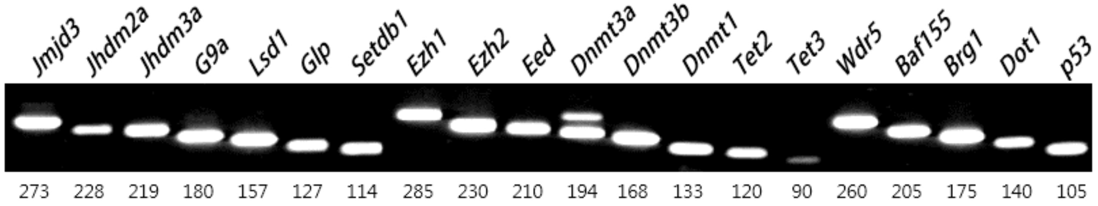

Fig. S4. Single-gene RT-PCR using cDNA from J1 mESCs.

Figure S6

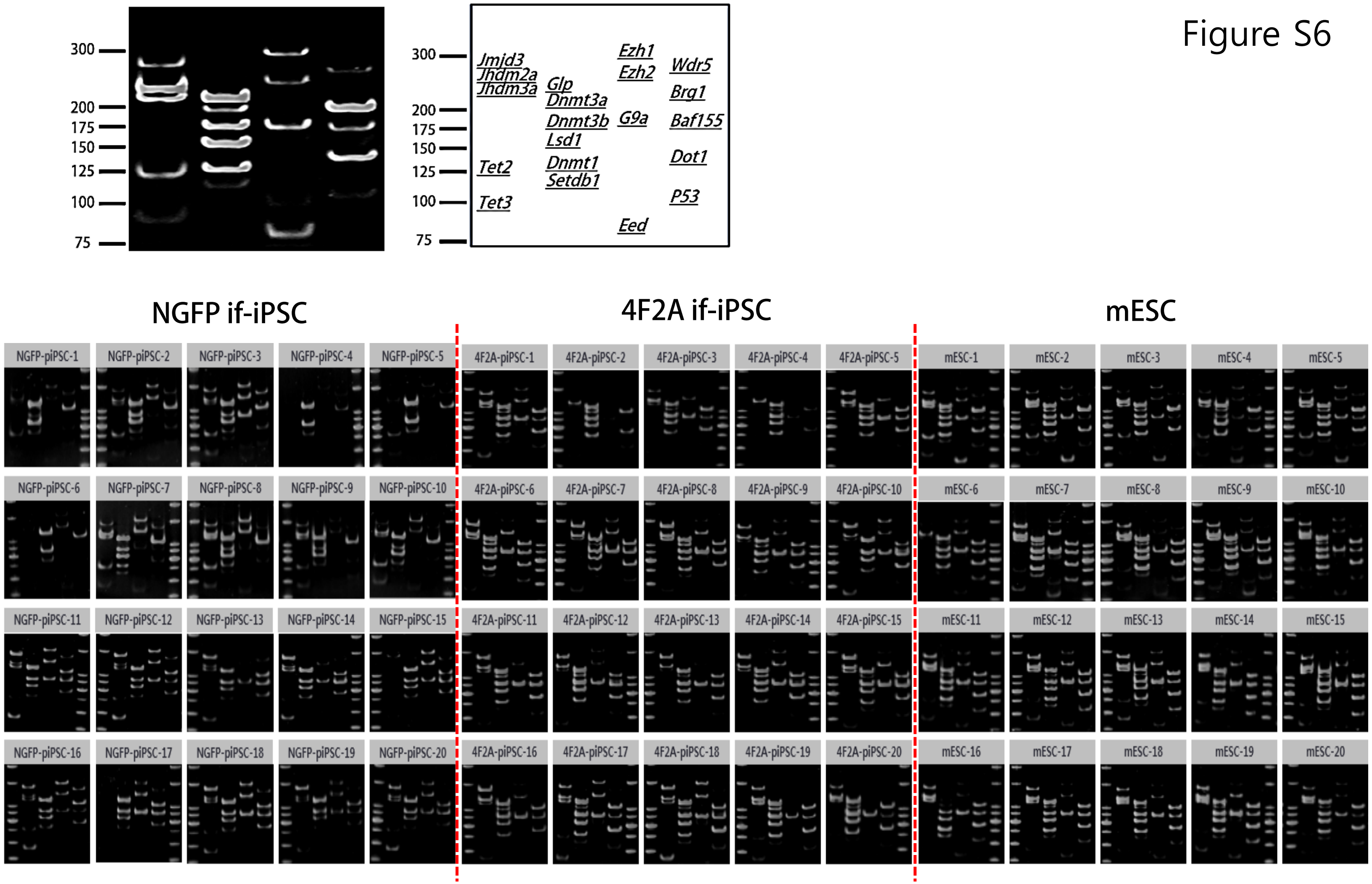

Fig. S5. Multiplex RT-PCR for reprogramming-related genes using single if-iPSC colonies.
